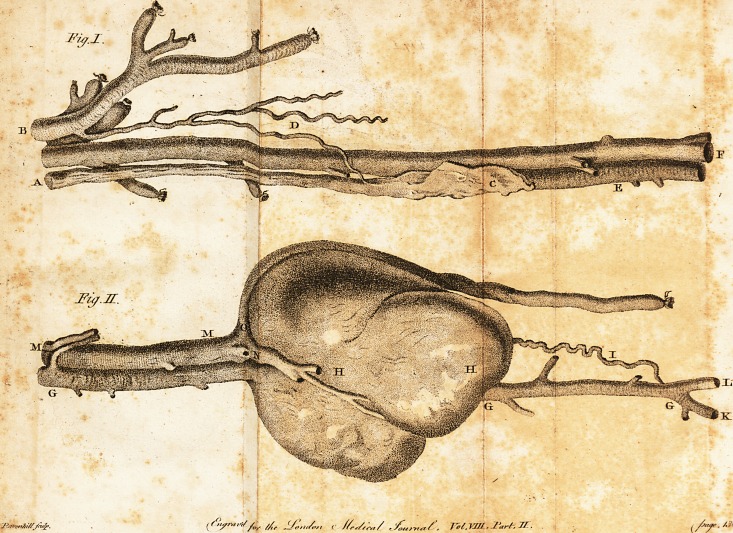# Supplement to the Account of Mr. Hunter's Method of Performing the Operation for the Popliteal Aneurism, Inserted in the Seventh Volume of This Work
*Page 391.


**Published:** 1787

**Authors:** Everard Home

**Affiliations:** Surgeon


*Fig. I.*

*Fig. II.*

IV. *Supplement to the Account of Mr.* Hunter'*s Method of performing the Operation for the Popliteal Aneurifm, inferted in the Seventh Volume of this Work *. Communicated in a fecond Letter to Dr.* Simmons, *by Mr.* Everard Home, *Surgeon, F. R. S.*

SOME time fince I fent you an account of a new mode of performing the operation for the popliteal aneurifm, by Mr. Hunter, at St. George's Hofpital; the man having lately died of a fever, afforded an opportunity of afcertaining the confequences of the operation and the ftate of the parts after the recovery, which, being all taken together, render the cafe very complete and fatisfactory; and the cafe being publifhed in the Medical Journal, I fend you the following account as a continuation of it.

The man was thirty-five years old at the time he underwent the operation, which was in December, 1785. In July, 1786, he was perfectly well, and returned to the driving of a hackney coach, which was his employment. From expofure to the weather, more particularly at night, he became fubject to repeated

* Page 391.

attacks attacks of cold, and, in March, 1787, was ſeized with a fever of the remittent kind which carried him off. During all this time, the limb on which the operation had been performed, was not at all affected.

He died on the 1ſt of April, 1787, and leave was procured, with ſome trouble, and conſiderable expence, for Mr. Hunter to examine the limb ſeven days after death, at which time it was entirely free from putrefaction.

The cicatrix, on the anterior part of the thigh, was ſcarcely diſcernible, but the parts under it felt hard. The ham had no appearance of tumour, and was, to the eye, exactly like that of the other limb; to the feel, however, there was a ſolid tumour filling up the hollow between the two condyles of the thigh bone.

The femoral artery and vein were taken out above the giving off the branch called profunda, and a little lower than the diviſion into the arteriæ tibiales and interoſſea, a portion of theſe branches being preſerved. The arteries and veins, that were pervious, being injected, the whole was carefully diſſected; and the following is an account of the appearances.

The femoral artery was impervious from the giving off the profunda, as low as the part which was included in the ligature, and at this part there was an offification, for about an inch and an half along the courfe of the artery, of an oval form, the rim of which was folid, becoming thinner towards the center, and not bony, but only ligamentous: below this part the femoral artery was pervious down to the aneurifmal fac, and contained blood, but did not communicate with the fac itfelf, having become impervious juft at the entrance.

What remained of the aneurifmal fac was fomewhat larger than a hen's egg, but more oblong, and a little flattened, extending along the fide of the artery below for fome way; the blood preffing with greater force in that direction, and diftending that part, fo as, in fome meafure, to give the appearance of a feparate bag. The fac was perfectly circumfcribed, not having the fmalleft remains of the lower orifice from the popliteal artery; whether this arofe from the artery being preffed upon by the inferior portion of the fac, as appears to be the cafe in common, or was in confequence of the fac contracting after the operation, I will not pretend to determine. It contained a folid coagulum of blood, which adhered to its internal furface. The coagulum, having a fection made of it, appeared to be compofed of concentrated lamellæ uniform in colour and confiftence.

The popliteal artery, a little way below the aneurifmal fac, was joined by a fmall branch very much contorted, which muft have arifen either from the profunda or the trunk of the femoral artery. About two inches below the fac the popliteal gave off or divided into the tibials.

The profunda was of the ufual fize, but a good deal offified for fome length after leaving the femoral artery: the two tibials, where they go off from the popliteal, were in the fame ftate.

The trunk of the femoral vein, where it paffed along the fide of the tumour, muft have been obliterated; for at this part it appeared to fend off three equal-fized branches, paffing over different parts of the aneurifmal fac: thefe muft have been dilated branches, none of them having the courfe which the trunk of the vein fhould have purfued.

Thefe appearances throw fome light on the changes which took place in the limb after the operation. A ligature being made upon the femoral artery, impeded the paffage of the blood into the fac fo much, as to allow it, in fome meafure, to collapfe, and its contents to coagulate, which rendered the opening of the artery into the ſac impervious; ſo that not only a ſtop was put to the increaſe of the tumour, but it muſt of neceſſity have become gradually more ſolid, and ſmaller, in conſequence of abſorption, till reduced to the ſize met with in the dead body.

The material conſequences taken notice of, coincide with the idea Mr. Hunter had formed prior to the operation.

The concluſion to be drawn from the above account appears to me a very important one, viz. That the ſimply taking off the force of the circulation from the aneuriſmal artery is ſufficient to effect a cure of the diſeaſe, or at leaſt to put a ſtop to its progreſs, and leave the parts in a ſtate from which the actions of the animal œconomy are capable of reſtoring them to a natural one.

In confirmation of this account, that the cure of an aneuriſm depends on taking off the force of the circulation, I ſhall mention a caſe that recovered without any aſſiſtance from art, and which I conſider to have got well upon the ſame principle. This caſe was more particularly under the care of Mr. Ford, Surgeon, in Golden Square, who will, I hope, lay a particular account of it before the public; I mean mean to notice it no farther than by endeavouring to account for the recovery, which may be explained by Mr. Hunter's obſervations on mortification.

The aneuriſm was in the femoral artery, and the ſwelling appeared upon the anterior part of the thigh a little above the middle, extending upwards, as it increaſed in ſize, nearly to the brim of the pelvis. Every attempt towards a permanent compreſſion of the artery above the tumour, juſt as it paſſes over the brim of the pelvis, proved ineffectual: the tumour enlarged to a very conſiderable ſize; a great degree of inflammation and ſwelling took place in the ſac and common integuments; and mortification appeared to be taking place in the ſkin which lay over it: while in this ſtate, the pulſation, before very evident in every part of the tumour, was no longer to be felt, nor even in the artery immediately above it; ſo that the ſteps preceding mortification had certainly taken place, the blood in the artery above having coagulated *; and this circum-

* In thoſe patients who die in conſequence of mortification of any part of the body, the artery leading to that part is found always completely ſtopped up for ſeveral inches in length

 by circumſtance was ſufficient to prevent the abſolute mortification coming on; for the artery above becoming impervious, put a ſtop to the dilatation of the ſac and all its conſequences.

From the time the pulſation ſtopped, the ſwelling and inflammation ſubſided, although exceedingly ſlowly, and the tumour diminiſhed, becoming more firm and ſolid, and at the time of writing this paper is very much reduced in ſize, and to the feel reſembles that found in the ham of the patient who is the ſubject of this paper.

Having in my former paper taken notice of an unſucceſsful caſe in which this mode of performing the operation for the aneuriſm was in ſome meaſure adopted at St. Thomas's hoſpital, I feel myſelf more particularly called upon at preſent to do away any cenſure that may have fallen upon this operation from an unſucceſsful caſe at St. Bartholomew's hoſpital, which has been the ſubject of medical converſation. I ſhall mention the operation, at which I was preſent, and give the reſult as briefly as I am able.

by a firm coagulum: this muſt take place prior to the mortification, and ſeems intended, for the wiſeſt purpoſes, to prevent hæmorrhage. *Taken from Mr. Hunter's Lectures.*

The

The aneurifm was in the ham, and the operation was performed by Mr. Pott in the following manner:

An incifion was made above the tumour, through the integuments, in the direction of the thigh between the two hamftrings, for about five inches in length; he then diffected down to the veffels at the upper end of the incifion, which being there deep feated, it proved both tedious and difficult. Having come down to the veffels, a double ligature was paffed, and the two portions tied feparately at nearly half an inch diftance. The depth of the incifion made it difficult for any but the operator and thofe immediately affifting him to fee what was included in the ligature, and no doubt was made at the time of its being any thing but the artery. The wound was dreffed up in the common way.

The fecond day after the operation a pulfation was felt in the tumour, which afterwards enlarged fo much, that Mr. Pott amputated the limb.

On diffection, the aneurifm did not appear to be in the artery which was included in the ligature, but was fuppofed to be in an anaftomofing branch.

I ſhall not go farther into the operation than as it applies to Mr. Hunter's mode of performing it, which leads me to the following remarks: — That from analogy the pulſation ſhould not have been felt in the tumour, had it been either in the trunk of the artery, or in an anaſtomoſing branch, if the popliteal artery above was rendered impervious; and if the branch diſeaſed went off from the femoral artery above the ligature, the pulſation ſhould have continued after the operation, and ſhould have been rendered more violent by it, which does not appear to have been the caſe; and farther, that the taking up the artery in the ham was taking it up under every diſadvantage reſpecting the future ſucceſs of the operation, either from the artery itſelf being diſeaſed, or the tumour being ſo contiguous to the violence done in the operation, that the whole ſac moſt probably would be affected by the conſequent inflammation, which ſeemed in ſome meaſure to have been the caſe, as I underſtand two abſceſſes were formed cloſe to the ſides of the ſac.

*Green Street,*

*Leiceſter Fields,*

May 23, 1787.

EXPLA-

### Explanation of the Plate.

The plate ſhews the femoral artery and vein injected and diſſected.

### Figure I.

The femoral artery, after it has paſſed through Poupart's ligament, divided below the giving off the profunda branch.

A, The trunk of the femoral artery impervious.

B, The profunda branch.

C, The portion included by the ligature, in the operation, in an oſſified ſtate.

D, An anaſtomoſing branch from the profunda communicating with the femoral artery.

E, The femoral artery below the ligature in a natural ſtate.

FF, The femoral vein.

### Figure II.

GGG, The continuation of the femoral artery.

HH, The remains of the tumour, the full ſize of the aneuriſmal ſac at the time of the patient's death.

I, An

I, An anaſtomoſing branch either from the profunda or femoral artery above.

K and L, The diviſion of the popliteal into the two tibial arteries.

MM, The continuation of the femoral vein.

N and O, Two enlarged branches going over the tumour in different directions.

## Figures and Tables

**Fig. I. Fig. II. f1:**